# Effect of Coenzyme-Q10 on Doxorubicin-Induced Nephrotoxicity in Rats

**DOI:** 10.1155/2012/981461

**Published:** 2012-12-17

**Authors:** Azza A. K. El-Sheikh, Mohamed A. Morsy, Marwa M. Mahmoud, Rehab A. Rifaai, Aly M. Abdelrahman

**Affiliations:** ^1^Department of Pharmacology, Faculty of Medicine, Minia University, 61511 El-Minia, Egypt; ^2^Department of Histology, Faculty of Medicine, Minia University, 61511 El-Minia, Egypt

## Abstract

Nephrotoxicity is one of the limiting factors for using doxorubicin (Dox) as an anticancer chemotherapeutic. Here, we investigated possible protective effect of coenzyme-Q10 (CoQ10) on Dox-induced nephrotoxicity and the mechanisms involved. Two doses (10 and 100 mg/kg) of CoQ10 were administered orally to rats for 8 days, in the presence or absence of nephrotoxicity induced by a single intraperitoneal injection of Dox (15 mg/kg) at day 4 of the experiment. Our results showed that the low dose of CoQ10 succeeded in reversing Dox-induced nephrotoxicity to control levels (e.g., levels of blood urea nitrogen and serum creatinine, concentrations of renal reduced glutathione (GSH) and malondialdehyde, catalase activity and caspase 3 expression, and renal histopathology). Alternatively, the high dose of CoQ10 showed no superior nephroprotection over the low dose, as there were no significant improvements in renal histopathology, catalase activity, or caspase 3 expression compared to the Dox-treated group. Interestingly, the high dose of CoQ10 alone significantly decreased renal GSH level as well as catalase activity and caused a mild induction of caspase 3 expression compared to control, probably due to a prooxidant effect at this dose of CoQ10. We conclude that CoQ10 protects from Dox-induced nephrotoxicity with a precaution to dosage adjustment.

## 1. Introduction

Doxorubicin (Dox), also known as adriamycin, is a broad spectrum anticancer anthracycline antibiotic that has been successfully used in treatment of a variety of hematological malignancies and solid tumors. Unfortunately, the use of Dox has been limited by the occurrence of dose-dependent toxicities to vital organs, as the heart, the kidney, and the liver [1]. The exact mechanism of Dox-induced nephrotoxicity is not yet completely understood. Renal Dox-induced toxicity may be part of a multiorgan damage mediated mainly through free radical formation eventually leading to membrane lipid peroxidation [2]. Induction of apoptosis and modulation of nitric oxide (NO) [3] are other mechanisms that may be involved in toxic adverse effects associated with Dox therapy. In addition, Dox may induce nephrotoxicity through its direct renal damaging effect, as it accumulates preferentially in the kidney [4]. Dox toxic effects to other organs as the heart and the liver may modulate blood supply to the kidney and alter xenobiotic detoxification processes, respectively, thus indirectly contributing to Dox-induced nephropathy.

A number of antioxidant compounds have been proposed as chemopreventive therapy for Dox-induced toxicity [5]. Of these compounds, the antioxidant coenzyme-Q10 (CoQ10) has been tried to minimize cardiotoxicity related to Dox therapy [6], but its effect on Dox-induced nephrotoxicity has not yet been elucidated. CoQ10, also known as ubiquinone, is the only naturally-occurring lipid soluble antioxidant that is endogenously synthesized [7]. Meat, fish, nuts, and certain oils are some of the richest nutritional sources of CoQ10, while much lower levels can be found in most dairy products, vegetables, fruits, and cereals [8]. It is used as a dietary supplementation and as a cotherapy in conjunction with medication in a number of conditions, including cardiovascular diseases, cancer, muscular neurodegenerative disorders, and diabetes [9].

The nephroprotective effect of CoQ10 is still controversial. On one hand, CoQ10 showed nephroprotective effects in some animal models [10, 11]. On the other hand, no renal protection has been reported in another animal study [12]. Furthermore, a study conducted on renal transplant recipient patients showed that despite the evident antioxidant effect of CoQ10, the kidney function reflected by creatinine level was not improved [13]. In the present work, an attempt was made to investigate the effect of CoQ10 on renal damage induced by Dox therapy.

## 2. Materials and Methods

### 2.1. Chemicals

CoQ10 powder was a generous gift from Mepaco (Egypt). Dox hydrochloride 10 mg vial (Pharmacia Italia, SPA, Italy), polyclonal rabbit/antirat caspase 3 antibody (1 mg/mL; Lab Vision, USA), biotinylated goat antirabbit secondary antibody (Transduction Laboratories, USA), kits for total protein concentration (Diamond diagnostics, Egypt), blood urea nitrogen (BUN), creatinine, reduced glutathione (GSH), and catalase (Biodiagnostic, Egypt) were purchased.

### 2.2. Animals and Experimental Design

Adult male Wistar rats weighing 185–250 g were obtained from the National Research Centre, Giza, Egypt. Animals were kept in standard housing conditions (12 h lighting cycle and 24 ± 2°C temperature), three or four rats/cage, and were left to acclimatize for one week. Rats were supplied with laboratory chow and tap water ad libitum. This work was ethically approved by the members of the board of the Faculty of Medicine, Minia University, Egypt (7/2010) in accordance with the EEC Directive of 1986 (86/609/EEC). Animals were randomly assigned to different experimental groups with no statistically significant difference in weight between groups. Animal groups were control-untreated group (*n* = 7), CoQ10L group (*n* = 7) treated with low (L) dose of CoQ10 of 10 mg/kg orally [10], CoQ10H group (*n* = 7) treated with high (H) dose of CoQ10 of 100 mg/kg orally [14], Dox-treated group (*n* = 15) receiving a single ip injection of Dox in a dose of 15 mg/kg (the dose was selected based on our preliminary experiments and a previous study by Ajith et al. [15] as renal toxicity was not seen at lower doses) given 5 days before animal sacrifice, Dox/CoQ10L and Dox/CoQ10H groups (*n* = 12 each) receiving similar Dox treatment, together with similar low or high doses of CoQ10, respectively, for 8 consecutive days, starting 3 days prior to Dox injection. Larger numbers of animals were assigned for groups receiving Dox, as higher rate of mortality was anticipated based on our preliminary experiments. CoQ10 powder, prepared in 1% carboxymethylcellulose, was administered by stomach tube. Animal not receiving CoQ10 received the same volume of 1% carboxymethylcellulose. Similarly, animals not receiving Dox were injected with the same volume of distilled water ip (Dox vehicle).

### 2.3. Evaluation of Renal Function

After 5 days of Dox injection, each rat was weighed then sacrificed by cervical dislocation. Venous blood samples were collected from the jugular vein, centrifuged at 5000 rpm for 15 min (Janetzki T30 centrifuge). As a marker of renal function and nephrotoxicity, BUN and serum creatinine were determined using colorimetric diagnostic kits according to the manufacturer's instructions.

### 2.4. Renal Homogenate Preparation and Determination of Protein Concentration

After sacrifice, both kidneys were rapidly excised and weighed. A longitudinal section of the left kidney was fixed in 10% formalin then embedded in paraffin for histopathological and immunohistochemical examinations. The rest of the kidneys were snap frozen in liquid nitrogen and kept at −80°C. For preparing renal tissue homogenate for biochemical analysis, kidney was homogenized (Glas-Col homogenizer), and a 20% w/v homogenate was prepared in ice-cold phosphate buffer (0.01 M, pH 7.4). The homogenate was centrifuged at 3000 rpm for 20 min, and the supernatant was kept at −80°C till used. Protein concentration was determined in the supernatant by total protein kit using spectrophotometer (Beckman DU-64 UV/VIS).

### 2.5. Evaluation of Renal GSH and Catalase Levels

Evaluation of renal antioxidant defense mechanisms was done by assessment of renal tissue GSH and catalase enzyme levels. For GSH, a spectrophotometric kit was used. Briefly, the method is based on that the sulfhydryl group of GSH reacts with 5,5′-dithio-*bis*-2-nitrobenzoic acid (Ellman's reagent) and produces a yellow colored 5-thio-2-nitrobenzoic acid which was measured colorimetrically at 405 nm using Beckman DU-64 UV/VIS spectrophotometer. Results were expressed as *μ*mol/g renal protein. Assessment of renal homogenate catalase antioxidant enzyme activity was determined from the rate of decomposition of H_2_O_2_ at 510 nm after the addition of tissue homogenate as described by colorimetric kit. The results were expressed as unit/g renal protein.

### 2.6. Assessment of Renal Lipid Peroxides and NO Levels

Renal lipid peroxidation was determined as thiobarbituric acid reacting substance and is expressed as equivalents of malondialdehyde (MDA), using 1,1,3,3-tetramethoxypropane as standard [16]. Results were expressed as nmol/g renal protein. The assessment of stable oxidation end products of NO, nitrite, and nitrate served as an index of NO production. This method was based on Griess reaction [17] that depends on the spectrophotometric measurement of total nitrites at 540 nm after the conversion of nitrate to nitrite by copperized cadmium granules. Results were expressed as nmol/100 mg renal protein.

### 2.7. Histopathological and Immunohistochemical Examination

Renal tissue that fixed in 10% formalin and embedded in paraffin were sectioned by a microtome at 5 *μ*m thickness and stained with hematoxylin and eosin for routine histopathological assessment. Three slides from each animal group, each with three sections, were subjected to semiquantitative microscopical analysis using light microscopy (Olympus CX41). Renal changes were graded as mild, moderate, or severe. Scores +, ++, and +++ are mild, moderate, and severe levels, revealing less than 25, 50, and 75% histopathological alterations of total fields examined, respectively.

Immunohistochemical staining was performed for caspase 3 using polyclonal rabbit/antirat caspase 3 antibody. Briefly, sections were deparaffinized, hydrated then washed in 0.1 M phosphate buffer. Sections were then treated with 0.01% trypsin for 10 min at 37°C then washed with phosphate buffer for 5 min. Endogenous peroxidases were quenched by treatment with 0.5% H_2_O_2_ in methanol, and nonspecific binding was blocked by normal goat serum diluted 1 : 50 in 0.1 M phosphate buffer. Tissues were incubated in the primary antibody (caspase 3; 1 : 1000) overnight at 4°C. Afterwards, tissues were washed and incubated in biotinylated goat antirabbit secondary antibody (1 : 2000) for 30 min. Following further 30 min incubation in vectastain ABC reagent, the substrate diaminobenzidine was added for 6 min, which gives brown color at the immunoreactive sites.

### 2.8. Statistical Analysis

Data was analyzed by one way ANOVA followed by Dunnett Multiple Comparison Test. The values are represented as means ± SEM. Chi-square test was used to analyze the significance of animal mortality results. All statistical analysis was done using GraphPad Prism software (version 5). The differences were considered significant when the calculated *P* value is less than 0.05.

## 3. Results

### 3.1. Effect of CoQ10 on Mortality and Kidney/Body Weight Ratio in Dox-Treated Rats

At sacrifice time, no mortality was observed in animals of control, CoQ10L, and CoQ10H groups (Table 1). On the other hand, Dox treatment significantly increased animal mortality. Coadministration of CoQ10 in both Dox/CoQ10L and Dox/CoQ10H groups did not result in statistically significant improvement in mortality. Kidney/body weight ratio was not affected by sole administration of CoQ10 in low or high dose. Treatment with Dox significantly increased the kidney/body weight ratio, which was not changed by administration of either doses of CoQ10.

### 3.2. Effect of CoQ10 on BUN and Creatinine in Dox-Treated Rats

Results of BUN and creatinine are summarized in Table 1. Rats receiving a single dose of Dox (15 mg/kg, ip) showed significant increase in BUN and creatinine levels compared to control group. Concomitant CoQ10 in low dose with Dox resulted in significant reduction of BUN and creatinine to levels comparable to normal controls. On the other hand, the high dose of CoQ10 resulted in less improvement of BUN and creatinine levels that were significant from Dox-treated group, but were still significantly higher from control. Neither the low nor the high CoQ10 alone, without Dox treatment, had any effect on these two markers of renal function compared to control.

### 3.3. Effect of CoQ10 on Renal GSH, Catalase, Lipid Peroxidation, and NO Levels in Dox-Induced Nephrotoxicity

**Figure 1:**
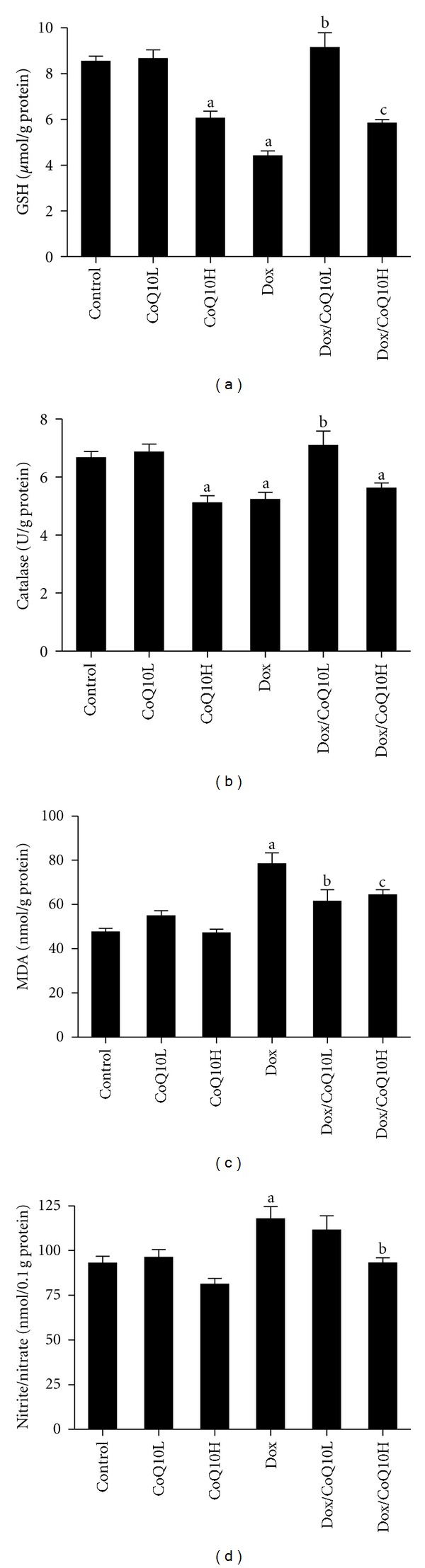
Effect of low and high doses of coenzyme Q10 (CoQ10) on renal (a) reduced glutathione (GSH), (b) catalase, (c) malondialdehyde (MDA), and (d) nitric oxide (nitrite/nitrate) levels in rats exposed to doxorubicin- (Dox-) induced nephrotoxicity. Animal groups tested are control-untreated group, animals treated with low or high dose CoQ10 alone (CoQ10L or CoQ10H, resp.), and animals treated with Dox or with Dox together with low or high CoQ10 dose (Dox/CoQ10L or Dox/CoQ10H, resp.). Values are represented as means ± SE of 6–11 observations. ^a^Significant difference compared to control, ^b^significant difference compared to Dox, without significant difference from control, and ^c^significant difference compared to Dox, with significant difference from control. Significant difference is reported when *P* < 0.05.

Treatment with Dox caused significant decrease in renal GSH and catalase levels compared with untreated control (Figures 1(a) and 1(b), resp.). Concomitant treatment of Dox with the low dose of CoQ10 restored renal GSH and catalase values to levels statistically comparable to control. On the other hand, concomitant treatment of Dox with the high dose of CoQ10 had no effect on renal catalase level, with less improvement on renal GSH that was significantly higher than Dox group but still significantly lower than control. The high dose of CoQ10, without Dox treatment, showed significant decrease of renal GSH and catalase compared to control.

Renal MDA was evaluated as an indicator of kidney lipid peroxidation (Figure 1(c)) and nitrite/nitrate ratio as an indicator of renal NO levels (Figure 1(d)). Dox significantly increased renal MDA and nitrite/nitrate ratio compared to control. Administrating CoQ10 in the low dose to Dox-treated animals retrieved MDA to levels statistically insignificant from control but had no effect on nitrite/nitrate ratio. On the other hand, giving CoQ10 in the high dose to Dox-treated animals improved MDA compared to Dox group but was still statistically significant from control and restored nitrite/nitrate ratio to levels comparable to that of control. CoQ10 alone in the low or the high dose had no significant effect on either renal MDA or NO levels.

### 3.4. Effect of CoQ10 on Renal Histopathology in Dox-Treated Rats

**Figure 2:**
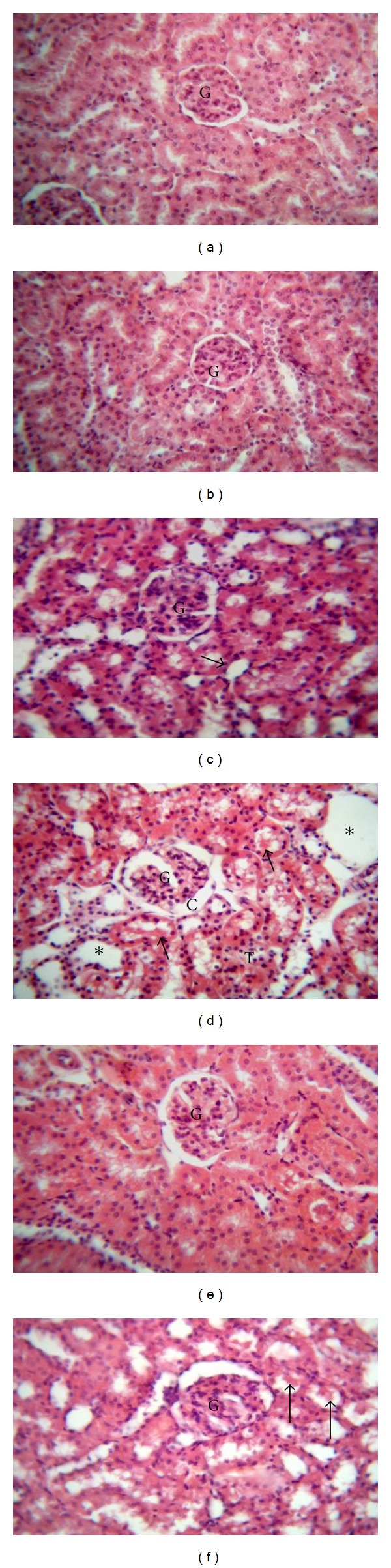
Effect of coenzyme Q10 (CoQ10) on kidney histopathological picture of doxorubicin- (Dox-) treated and untreated rats. A photomicrograph of a section in rat kidney (H and E ×400) of ((a) and (b)) untreated control and low dose CoQ10 treated (CoQ10L) groups, respectively, with normal structure of renal glomeruli (G) and cortical tubules; (c) high dose CoQ10 treated (CoQ10H) group with normal renal glomeruli (G), but mild degeneration of the epithelial lining of some tubules (arrow); (d) Dox-treated group with dilated Bowman's space (c), severe degenerative changes observed in the renal tubules with exfoliated cells (T). Some tubules are filled with protein casts (arrows) and some showing cystic dilatation (stars); (e) Dox/CoQ10L group with regeneration of renal tubular epithelial cells and normal morphology of renal cortex and glomeruli (G); (f) Dox/CoQ10H group with marked degeneration of renal tubules with exfoliated epithelial cells and casts (arrows).

Histopathological examination revealed that control and CoQ10L groups had normal structure of renal glomeruli and cortical tubules (Figures 2(a) and 2(b); Table 2). On the other hand, Dox-treated group presented with dilated Bowman's space and marked degeneration of renal tubules that showed exfoliated cells, protein casts, and cystic dilatation (Figure 2(d)). Concomitant administration of CoQ10 in the low dose with Dox resulted in reversal of histopathological damage induced by Dox, with regeneration of renal epithelial cells lining of cortical tubules and restoration of normal morphology to renal cortex (Figure 2(e)). The high dose of CoQ10 given with Dox, however, did not reverse morphological changes seen in Dox group, but showed marked degeneration of renal tubules with exfoliated epithelial cells and casts comparable to Dox group (Figure 2(f)). Furthermore, the high dose without Dox treatment in CoQ10H group showed degeneration of the epithelial lining of some tubules (Figure 2(c)).

### 3.5. Effect of CoQ10 on Renal Apoptosis in Dox-Induced Nephrotoxicity

**Figure 3:**
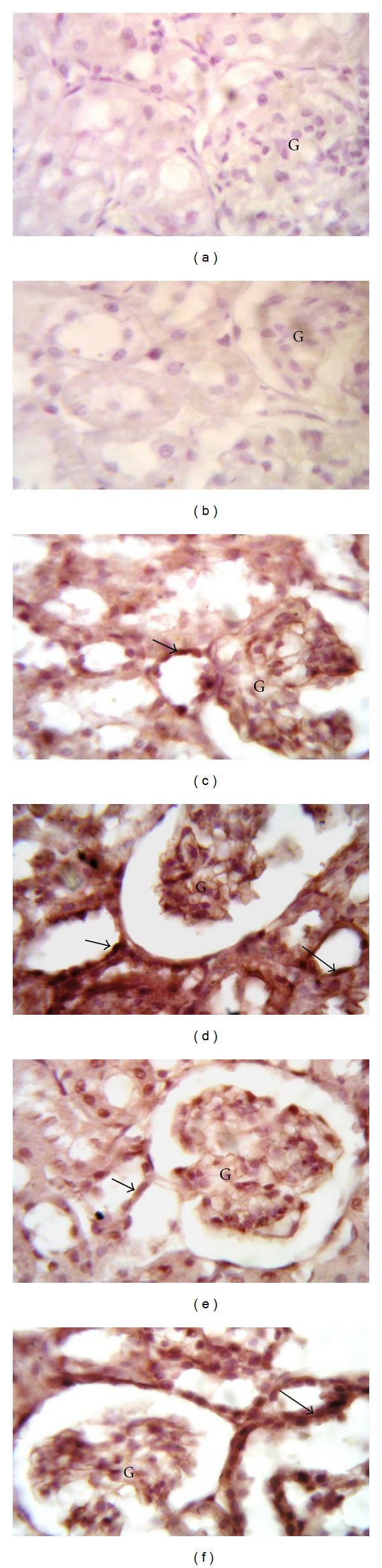
Effect of coenzyme Q10 (CoQ10) on caspase 3 immunohistochemical staining of doxorubicin- (Dox-) treated and untreated rat kidney. Localization of caspase 3 immunoreactivity in the kidney cortex (×1000) of ((a) and (b)) untreated control and low dose CoQ10 treated (CoQ10L) groups, respectively, showing negative immunoreactivity; (c) high dose CoQ10 treated group (CoQ10H) showing faint expression within the glomeruli (G) and the renal tubules (arrow); (d) Dox-treated group showing high expression in the renal glomeruli (G) and renal tubules. The expression is mainly cytoplasmic, but with some nuclei showing positive expression (arrows); (e) Dox/CoQ10L group showing faint expression within the glomeruli (G) and the renal tubules (arrow); (f) Dox/CoQ10H group showing high expression in the glomeruli (G) and the renal tubules (arrow).

**Figure 4:**
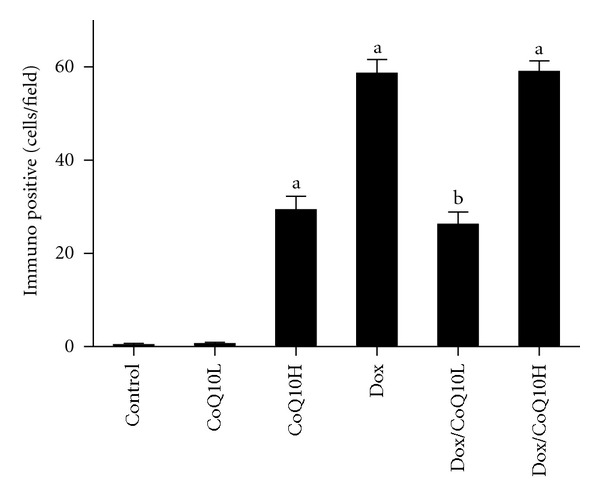
Effect of two doses of coenzyme Q10 (CoQ10) on renal caspase 3 immunohistochemical semiquantitative analysis in rats exposed to doxorubicin- (Dox-) induced nephrotoxicity. Kidneys were isolated from control untreated group, animals treated with low or high dose CoQ10 (CoQ10L or CoQ10H, resp.), and animals treated with Dox, or with Dox together with CoQ10L or CoQ10H, respectively. Values are represented as means ± SE of number of immuno-positive cells for caspase 3 in sections of 3 animals of each group, 5 fields/section. ^a^Significant difference compared with control and ^b^significant difference compared to Dox, with significant difference from control. Significant difference is reported when *P* < 0.05.

As a marker of apoptosis, induction of caspase 3 was evaluated by immunohistochemical staining (Figure 3). Semiquantitative analysis was further performed to calculate the degree of significance (Figure 4). Immunohistochemical staining of rat kidney showed that administration of Dox caused significant increase in the immunoreactivity of caspase 3 compared to control, which was highly expressed in renal glomeruli and tubules both cytoplasmically and in some nuclei (Figure 3(d)). Concomitant administration of CoQ10 in the low dose with Dox significantly decreased caspase 3 expression to levels significant from Dox alone (Figure 3(e)). On the other hand, the high dose of CoQ10 with Dox failed to produce a similar effect, as it showed high caspase 3 expression in the glomeruli and renal tubules (Figure 3(f)). Interestingly, administration of the high dose of CoQ10, but not low dose, without Dox caused significant expression of caspase 3 compared to control (Figure 3(c)).

## 4. Discussion

Despite the extensive clinical utilization of Dox in the treatment of cancer patients, the mechanism by which it produces its nephrotoxic adverse effect is still under intense debate. One of the mechanisms suggested is free radical formation and oxidative stress [1]. The level of the endogenous antioxidant CoQ10 seems to increase in human plasma after Dox therapy [18]. This is probably through upregulation of CoQ10 gene expression as a cellular defense mechanism against chemotherapy to promote cell survival [19]. This directed our attention to investigate the role of CoQ10 as a possible nephroprotective agent against Dox-induced renal damage, especially after its success in protecting from Dox-induced cardiotoxicity [6].

The dose of Dox used in this study corresponds to the dose that is currently being used in clinical practice [20]. In the present study, this dose produced acute renal function deterioration in the animal group receiving it. Such alteration in renal function was completely restored to levels statistically insignificant from control by prophylactic coadministration of CoQ10 in a low dose. The high dose of CoQ10 also improved Dox-induced renal function deterioration, but still significantly higher than control levels. This indicates that increasing CoQ10 dose does not confer more nephroprotection against Dox-induced renal damage.

Improvement of Dox-induced nephrotoxicity was previously tried by compounds that partially succeeded in preserving normal renal function and structure probably through their antioxidant effects, as caffeic acid phenethyl ester [21], Zingiber officinale Roscoe [15], and Solanum torvum [22]. Here, a prophylactic low dose of CoQ10, 3 days before and extending 5 days concurrently with Dox treatment, restored most of kidney antioxidant parameters and apoptotic signs to control levels. The enhanced renal antioxidant status resulting from low dose CoQ10 prophylactic treatment could explain its nephroprotective effect. Such nephroprotective effect is probably not accompanied by any alteration in Dox disposition, including metabolism, biliary excretion, and clearance [23], nor with deterioration in Dox antineoplastic properties as reported in breast cancer cell cultures [24].

Increasing the dose of CoQ10 given with Dox therapy did not show any improvement in renal function or histopathological structure over low CoQ10 dose. Furthermore, cotherapy of Dox with the higher CoQ10 dose resulted in disappointing effects concerning renal antioxidant status and apoptosis. These results imply a prooxidant effect of CoQ10 in the high dose. Indeed, some antioxidants were reported to possess prooxidant effects at higher doses, as the flavonoids: quercetin, myricetin, kaempferol [25], and curcumin [26] that were found to mediate induction of reactive oxygen species at high concentration. Some studies suggested a similar prooxidant effect for CoQ10 *in vitro* [27–29]. This was further supported by the prooxidant effects reported for the CoQ10 analog, known as mitochondrial-targeted coenzyme Q mitoQ [30]. A study conducted on renal hemodialysis patients showed that CoQ10 suppressed the oxidative stress and still, unexpectedly, decreased oxygen radical absorbing capacity [31]. In these patients suffering from diminished renal function, concentration of CoQ10 may be higher than normal, which probably resulted in the appearance of such prooxidant effect. Here, we provide for the first time a mechanistic proof of prooxidant mechanisms of high dose CoQ10 *in vivo*, which, when given alone, resulted in oxidative stress evident by decreased renal GSH and catalase levels and induced mild renal apoptosis implicated by renal caspase 3 expression.

In conclusion, at a dose of 10 mg/kg, CoQ10 protects against Dox-induced nephrotoxicity in rats. However, increasing the dose of CoQ10 concomitantly given with Dox to 100 mg/kg is not more nephroprotective. This is probably due to a prooxidant effect of CoQ10 manifested at the high dose and seen even when it is given alone without Dox.

## Figures and Tables

**Table 1:** Effect of coenzyme Q10 (CoQ10) on percent of animal mortality, kidney/body weight ratio, blood urea nitrogen (BUN), and creatinine in doxorubicin- (Dox-) induced nephrotoxicity in rats.

Groups	Mortality %	Kd/Wt	BUN (mg/dL)	Creatinine (mg/dL)
Control	0	5.9 ± 0.8	8 ± 1	0.95 ± 0.04
CoQ10L	0	5.9 ± 0.6	8 ± 2	0.9 ± 0.1
CoQ10H	0	6.4 ± 0.7	10 ± 1	0.9 ± 0.1
Dox	40^a^	7.2 ± 0.6^a^	358 ± 97^a^	2.6 ± 0.3^a^
Dox/CoQ10L	25	6.7 ± 0.8	31 ± 7^ b^	1.7 ± 0.1^b^
Dox/CoQ10H	8	6.8 ± 1.1	98 ± 31^c^	1.9 ± 0.2^c^

CoQ10L and CoQ10H are rats treated with low and high doses of CoQ10, respectively; Kd/Wt is kidney/body weight ∗ 1000 ratio. Values are representation of 6–11 observations as means ± SEM, except survival which is represented as a percentage. ^a^Significant difference compared to control, ^b^significant difference compared to Dox group, with no statistically significant difference compared to control, and  ^c^significant difference compared to Dox group, but with also significant difference compared to control group. Results are considered significantly different when *P* < 0.05.

**Table 2:** Effect of coenzyme Q10 (CoQ10) on severity of histopathological lesions in doxorubicin- (Dox-) induced nephrotoxicity in rats.

Groups	Tubular degeneration	Tubular dilatation	Dilated Bowman's space	Protein casts
Control	0	0	0	0
CoQ10L	0	0	0	0
CoQ10H	++	+	0	0
Dox	+++	++	+	++
Dox/CoQ10L	+	0	0	+
Dox/CoQ10H	+++	++	+	+

CoQ10L and CoQ10H are rat groups treated with low (L) or high (H) dose CoQ10, respectively. Score level 0 was considered normal. Scores +, ++, and +++ are mild, moderate, and severe levels, revealing less than 25, 50, and 75% histopathological alterations of total fields examined, respectively. Score represents values obtained from tissue sections of 3 animals of each group, 5 fields/section (×400).
